# Female Sex as a Thromboembolic Risk Factor in the Era of Nonvitamin K Antagonist Oral Anticoagulants

**DOI:** 10.1155/2020/1743927

**Published:** 2020-06-18

**Authors:** Mariacarla Gallù, Giulia Marrone, Jacopo Maria Legramante, Antonino De Lorenzo, Nicola Di Daniele, Annalisa Noce

**Affiliations:** ^1^UOC of Internal Medicine, Center of Hypertension and Nephrology Unit, Department of Systems Medicine, University Tor Vergata, Rome, Italy; ^2^PhD School of Applied Medical-Surgical Sciences, University Tor Vergata, Rome, Italy; ^3^Emergency Department University Tor Vergata, Rome, Italy; ^4^Section of Clinical Nutrition and Nutrigenomic, Department of Biomedicine and Prevention, University Tor Vergata, Rome, Italy

## Abstract

Sex-specific differences have been definitively demonstrated in cardiovascular (CV) diseases. These differences can also impact on the effects of CV therapies. Female sex is recognized as an independent predictor of thromboembolic risk, particularly in older patients. Most of strokes are due to atrial fibrillation (AF). Women affected by AF have higher stroke risk compared to men. The introduction of novel oral anticoagulants (NOACs) for long-term anticoagulation completely changed the anticoagulant therapeutic approach and follow-up of patients affected by nonvalvular atrial fibrillation (NVAF). CHA2DS2-VASc stroke risk scoring in use in the current international guidelines attributes 1 point to “female sex”. Besides, no anticoagulation is indicated for AF female patients without other risk factors. Interestingly, NOACs seem to normalize the differences between males and females both in terms of safety and efficacy, whereas residual higher stroke risk and systemic embolism persist in AF women treated with vitamin K antagonist anticoagulants VKA with optimal time in therapeutic range. Based on the CHA2DS2-VASc score, NOACs represent the preferred choice in NVAF patients. Moreover, complete evaluation of apparently lower risk factor along with concomitant clinical conditions in AF patients appears mandatory, particularly for female patients, in order to achieve the most appropriate anticoagulant treatment, either in male or in female patients. The present review was performed to review sex differences in AF-related thromboembolic risk reported in the literature and possibly highlight current knowledge gaps in prevention and management that need further research.

## 1. Introduction

There is no doubt that men and women are biologically different in terms of body weight, body surface area, total body water, the distribution of extracellular, and intracellular water, as well as differences in the response to drug treatments. Several possible potential reasons are represented by biological differences between men and women [1] such as coagulation mechanisms, in e.g., during different female hormonal status at various ages (menstrual cycle, pregnancy, postmenopause), endothelial function, oral contraceptive therapy, and hormone replacement therapy. The risk for ischemic stroke in women doubles between the ages of 55 and 65, coinciding with the menopausal period, when severe estradiol levels and estrogen receptor reduction occurs. This condition could also favour a hypercoagulable state also through an increased production of inflammatory cytokines [2]. Several prothrombotic biomarkers, such as D-dimer, von Willebrand factor, and beta-thromboglobulin are present in higher concentrations in patients affected by atrial fibrillation (AF) [3, 4].

Von Willebrand factor and soluble E-selectin are also known markers of endothelial dysfunction or damage, as well. Sex differences have been found in von Willebrand factor concentration, which is higher in women compared to males [5].

The hemorrhagic burden includes various factors involved in endothelial function, platelet aggregation, and vascular changes during different biological phases and coagulation factor activation.

Differences in the volume of distribution, larger “free fraction” of drugs, as well as differences in drug clearance, may lead to drug overexposure in women. Differences in receptor numbers, in receptor binding, and in the signal transduction pathway following receptor binding may all make women more sensitive to drug effects [6].

Differing gene expression directly reflects on vascular function, myocardial response to a stress condition, and sex-specific drug metabolism. These are all referred to as “sex differences” and may be reproduced in animal models.

Conversely, “gender characteristics” arise from sociocultural environments, different behaviours, nutrition, dietary habits, environmental exposure, attitudes towards lifestyle, and compliance to therapy. They are unique to the human. Both sex and gender are equally important for cardiovascular (CV) diseases. Some authors use sex/gender definition for medical differences between men and women [1, 7].

Women take a greater amount of medications compared to men, leading to a higher potential for drug interactions. In the setting of oral anticoagulation, the impact of sex on the effectiveness and safety of warfarin has been previously analysed, but not fully elucidated.

The ATRIA study [8], a large cohort prospective study, reported a similar rate of major bleeding between sexes, during warfarin treatment, even if women showed a lower risk of intracranial hemorrhage (ICH). Besides, women had higher rates of ischemic stroke and peripheral embolism while not taking warfarin, than men did.

In the last decade, much interest has been developing in sex and gender-specific aspects of many areas of medicine, in terms of pathophysiology, clinical manifestation, and management, as well. The European Union (EU) current research framework programme “Horizon 2020”, included “gender” into biomedical research as one of the most relevant requirements for a more accurate improvement of scientific quality and knowledge. As far as CV disease is concerned, biological differences between men and women reflect also on several CV clinical patterns and incidence, pathophysiology, response to treatment, and clinical outcomes. Such differences can affect prognosis, with important implications in terms of management and public health.

The present review was performed to analyse the influence of the female sex on AF-related thromboembolic risk in either non-anticoagulated AF patients or AF patients on vitamin K antagonist anticoagulants (VKAs) compared to Novel Oral Anticoagulants (NOACs). Female patients undergoing VKAs therapy exhibit a higher risk of stroke and systemic embolism (SE), despite an optimal time in therapeutic range (TTR), NOACs seem to resolve sex differences both in terms of safety and efficacy. Concern arises about the revised CHA2DS2-VASc risk stratification score, according to which, female sex is recognized as an independent predictor of thromboembolic risk. A higher incidence of stroke in female patients was reported by several authors [9–11]. Female sex was significantly associated with an increased incidence of stroke particularly among patients aged ≥75 years. Indeed, the 2012 edition of the European Society of Cardiology guidelines [12] suggested to apply one point only to females aged ≥65 years. Some authors [5] found a higher stroke incidence in women of all ages. Data on stroke risk of females aged 65-75 years are conflicting. Women with AF can be more symptomatic and present with more comorbidities because of the older age. The aim of the present review is to focus on the difference between male and female sex in terms of thromboembolic risk and in terms of therapeutic choice in order to highlight the need to offer proper diagnostic tools and management to both men and women [13].

## 2. Methods-Search Strategy

A literature search was conducted on PubMed, electronic database using the keywords “atrial fibrillation” [Title/Abstract] and “Novel Oral Anticoagulants” [Title/Abstract] or “NOACs” [Title/Abstract] or “sex”/“gender” [Title/Abstract] or “thromboembolic risk” [Title/Abstract].

Reference lists and related records were manually reviewed. The search was limited to English language papers published until January 20th, 2020.

## 3. Stroke and Atrial Fibrillation

Every year, about 17 million people all over the world die from CV diseases, heart attack, and stroke, about 5 million die from this disease [14]. The majority of strokes are ischemic-induced [15]. Patients undergoing AF are up to five-fold likely to suffer from ischemic stroke, compared to patients without AF, and a high percentage suffers for permanent disabilities. AF is the most common supraventricular arrhythmia in the world [16]. Although not directly life-threatening, it can precipitate acute heart failure, pulmonary edema, stroke, and sudden death. AF negatively influences the quality of life, has heavy implications on work activity, and increases the risk of hospitalization. Several differences in epidemiological patterns, clinical manifestations, and incidence of stroke have been reported between women and men, affected by AF, particularly in the elderly population. Aged women have higher blood pressure than men and a higher prevalence of heart failure with preserved ejection fraction [17]. As widely reported in the literature from epidemiological studies [16], AF incidence is progressively increasing and represents an independent risk factor for ischaemic stroke. A number of 2.7 million new cases *per* year for men and 2.0 million for women have been estimated [18, 19]. In the EU, 4-17 million AF patients are anticipated by 2030 with 120000-215000 newly diagnosed patients *per* year [13]. A 2.5-fold increase in the prevalence of AF is projected by the year of 2050 [17, 19].

A five-fold increase in CV accidents and systemic embolization has been demonstrated in patients with AF. Epidemiological studies highlighted a progressive global burden of AF incidence, with a significant impact on public health and subsequent increase of mortality related to it [13, 18, 20].

Several studies described sex differences in epidemiology and prognosis of AF, as widely demonstrated [15, 17], there is a significant association between stroke and women aged ≥75 years. Of note, patients aged <65 years, without any other risk factor, had low stroke rates, either males or females [21].

AF incidence increases with age (3.7%–4.2% of those aged 60–70 years, and 10%–17% of those aged >80 years) [17]. Moreover, women make up the greater proportion (60%) of AF patients who are over the age of 75 years, because of their longer lifespan. Women with AF have a higher mortality rate, even after adjustment for baseline comorbid conditions and treatment with anticoagulants [15].

Despite substantial advances in rhythm control therapy, anticoagulation plays a major role in AF treatment for the prevention of thromboembolic stroke [15].

## 4. Anticoagulation Benefit

Based on European and North American epidemiological studies, different prevalence and prognosis between men and women affected by AF have been largely demonstrated. While men have a higher risk of AF, female AF patients harbour a greater risk of having a stroke particularly in aged (≥75 years) patients and irrespectively of warfarin therapy [8, 15, 19, 21]. The risk of death in women with AF is similar or higher than that in men with AF. The Atrial Fibrillation Follow-up Investigation of Rhythm Management (AFFIRM) [22] found that women were more likely outside the TTR with consequent sub therapeutic international normalized ratios (INRs). Besides, those women with a comparably high TTR (>66%) still had significantly more ischemic strokes.

The largest prospective, observational, multicentre Global Anticoagulant Registry in the Field of AF (GARFIELD-AF) [15] explored the impact of sex on 1-year outcomes in patients with nonvalvular atrial fibrillation (NVAF). Outcomes of AF female patients markedly differ from men, with a higher incidence of stroke and higher mortality, even after adjusting for comorbidities. Notably, women were aged 75 or older at the time of diagnosis of NVAF, as reported from other studies [23]. Women population was more burdened by hypertension than men, whereas other risk factors, such as diabetes and prior stroke were similar in both sexes. Notably, there were small differences in stroke risk factors in men and women with newly diagnosed NVAF, beyond the impact of sex. The pattern of treatment was almost identical in the two groups (male and female).

Of a total of 63.8% women and 62.9% men receiving anticoagulant therapy, respectively, 46.8% and 46% received VKAs. The response to anticoagulant therapy was significantly more evident in men compared to women in terms of reduction of stroke/SE, similar for major bleeding and all-cause mortality.

The authors conclude that of the 28.624 patients enrolled, women showed a higher risk of stroke/SE and the reduction on event rates with AC therapy was less than in men.

The lower benefit on stroke rates in women has been partially explained by the lower weight and lower adherence to therapy, leading to wider variations in anticoagulation control and poorer anticoagulation in women compared to men [24].

Several reports actually describe differences in epidemiology, clinical patterns, and both thromboembolic and bleeding risk between males and females [17].

The possible existence of differences between males and females in thrombotic and haemorrhagic risk and in the outcomes during anticoagulant therapies may pose the question of whether any sex-specific management is required.

## 5. Female Sex as a Risk Factor

In the large observational ATRIA study on “gender differences in the risk of ischemic stroke and peripheral embolism in AF”, female patients while off-warfarin showed a higher risk of stroke, than did men, even when adjustment for clinical risk factors was performed. Both younger and aged female patients showed higher rates of thromboembolism. Similar rates of major bleeding were reported between sexes, whereas female patients presented a lower risk of ICH. The authors conclude that female sex is an independent risk factor for thromboembolism and should be taken into account for a correct anticoagulant therapeutic choice in patients affected by AF. These findings indicate that women with AF face a higher absolute risk for thromboembolism independently of other risk factors and should gain more from anticoagulant therapy [8]. Besides, no homogeneous evidence was previously reported in the literature and, if some studies found an increased risk in women, others were not able to confirm this finding and female sex as one of the independent risk factors for stroke [9] was not included in 2007 stroke risk score.

The systematic review and large meta-analysis carried out by Wagstaff et al. [10] included 17 studies, five randomized-controlled trials, and 12 observational studies on anticoagulated and non-anticoagulated AF patients. The analysis found that women carry a risk ratio of 1.31 (95% CI 1.18-1.46) for stroke, and the risk appears greater for women ≥75 years. The authors conclude that women affected by AF have increased risk of stroke, regardless of oral anticoagulation therapy. A correct and comprehensive evaluation of stroke risk should include female sex, as a risk factor in all AF patients.

In 2010, the stroke risk stratification score used in the international guidelines has been revised to the CHA2DS2-VASc score, for a more accurate evaluation of risk [12].

The revised CHA2DS2-VASc (congestive heart failure, hypertension, age ≥ 75 years “doubled”, diabetes, stroke “doubled”-vascular disease, age “65-74”, and sex “female”) score attributes 1 point to female sex. Female sex was finally accepted as an independent stroke risk factor [25], in that it increases the risk of stroke in older women, when other risk factors coexist. Moreover, it is considered as a “risk modifier” as it does not seem to increase stroke risk when no other risk factor is present [10].

According to the 2016 ESC Guidelines for the management of AF developed in collaboration with EACTS [13], anticoagulation is recommended with a 1 point or more “CHA2DS2-VASc” score for men and 2 or more for women. Moreover, anticoagulation is not indicated in women without other risk factors and receives only a Class IIa-LoEB recommendation when only one additional risk factor is present. The increased risk in women compared to men, is not yet completely understood.

## 6. From VKA to NOACs

After decades of VKAs, NOACs changed both the approach and the follow up of patients on anticoagulant therapy. Dabigatran, rivaroxaban, apixaban, and edoxaban have been studied in large randomized trials, in which they emerge as the preferred choice, particularly in naïve patients, as mentioned in the European Heart Rhythm Association (EHRA 2018) [26]. Physicians are getting more and more familiar with the use of these drugs in clinical practice, but many unresolved questions on how to optimally use these agents remain, especially in specific clinical situations. Very well-designed multicentre clinical trials (Table 1) on the effectiveness of the newer direct oral anticoagulants and VKAs were performed, but sex-associated risk, during NOACs treatment, is not completely understood.

Panchloy et al. conducted a large meta-analysis (sixty-four randomized studies), which addressed the issue of sex-related outcome differences in either warfarin or NOACs therapy in NVAF. The results indicated that AF female patients have a significantly higher residual risk of cerebrovascular accidents (CVA)/SE, when warfarin is prescribed, as compared to men. The authors conclude that sex difference disappears when a pooled population treated with NOACs is analysed. Moreover, they suggest an increased net clinical benefit of NOAC agents compared with warfarin in treating women with AF. Less major bleeding risk was observed in NOACs female patients compared to male patients [19].

The large meta-analysis by Poli and Antonucci [17] clearly established that women with AF carry a persistently higher stroke risk compared to men, even when adequate anticoagulation is prescribed. The higher stroke risk among AF women seems to be confirmed, although none of the phase III trials has been powered to determine a sex difference in the efficacy of NOACs [27–30]. However, the benefit of anticoagulation was similar between males and females (Table 2).

Reports from a large cohort study performed by Law et al., comparing NOACs vs warfarin efficacy and safety in men and women according to anticoagulation control (TTR), demonstrated comparable results of NOACs versus warfarin in male patients. NOACs treatment led to a lower risk of ICH and all-cause mortality, only in women. The association of lower ICH risk remained when compared to warfarin patients with good anticoagulation control [31].

A large systematic review of the available evidences from randomized trials on NOACs along with a systematic meta-analysis of the 4 phase III clinical trials was conducted by Proietti et al. [32]. The aim was to investigate sex differences in stroke/SE events and major bleedings for a better assessment of major adverse outcomes according to sex, during the treatment. Data was collected directly from the original papers with the results on dabigatran, rivaroxaban, apixaban, and edoxaban in NVAF. Interestingly, only minor differences were found either in efficacy or in safety between males and females when NOACs were prescribed. A higher risk was found for the occurrence of stroke/SE in males. No difference was found for major bleeding between male and female patients. In the paper by Avgil Tsadok et al. [33], dabigatran users (both dabigatran 110 mg. and dabigatran 150 mg) were matched with warfarin users among AF patients, stratified according to sex (50,4% females, 49,6% males). The mean CHA2DS2-VASc score was higher in females compared to males. Female patients were older and found more burdened with hypertension and a previous history of stroke/transient ischemic attack (TIA). Females were less likely prescribed dabigatran compared with warfarin and when dabigatran was chosen, they received dabigatran 110 mg. After a median follow up of 1.3 years, incidence rates of stroke did not differ between male and female patients, both for dabigatran 110 mg and dabigatran 150 mg.

Moreover, Vinereanu et al. conducted a subgroup analysis of the Apixaban for Reduction in Stroke and Other Thromboembolic Events in Atrial Fibrillation (ARISTOTLE) trial [34]. While women had more risk factors (age, hypertension, and previous thromboembolic events) and higher “CHA2DS2-VASc score” (as female sex scores 1 point), apixaban normalized the difference between males and females, when compared to warfarin. Notably, no differences in bleeding risk were described. Indeed, analysed data from the AVERROES (apixaban vs aspirin) trial [35] demonstrated that the use of Apixaban reduced the ischemic induced stroke and normalized the difference between males and females, who were even more burdened by other risk factors. Moreover, there was no difference in the reduction of stroke risk in comparison with aspirin, in both sexes. Data from “real life” about dabigatran use, even if female patients had, again, more risk factors, did not show any difference in the prevention of stroke/TIA occurrence. Additionally, a better safety profile was observed in women treated with apixaban [32]. Nonetheless, dabigatran showed better protection against bleeding events in male than in female patients, either with low or high dose dabigatran regimens [32]. A small advantage in terms of risk reduction was observed in women and data on bleeding demonstrated a better safety profile for female patients taking apixaban.

Preliminary data from the “Outcomes Registry for Better Informed Treatment of Atrial Fibrillation (ORBIT-AF) registry” [36], a multicentre, prospective, ambulatory-based registry of incident and prevalent AF, demonstrated that TTR in women is more often lower than in men, when warfarin is prescribed. Therefore, the elevated residual risk of CVA/SE observed in the female cohort has been possibly ascribed to this mechanism. This correlates to pharmacodynamics and pharmacokinetic advantages provided by NOACs, leading to a more stable anticoagulant effect, “superior” to warfarin [19].

Therefore, data from literature seem to actually highlight some differences basing on VKAs and NOACs treatment. On the other hand, as NOACs differ in pharmacologic properties, Moseley et al. performed an indirect comparison of the 4 NOACs for efficacy and safety, using warfarin as a comparator, and no apparent difference for any single agent between males and females [37] was demonstrated. Besides, the authors underline that a very large patient population is required to find statistically significant differences.

Another recent observational study conducted by Avgil Tsadok et al. [21], demonstrated that the reduced risk of ischemic stroke in patients on rivaroxaban compared to dabigatran and warfarin was observed in men, while bleeding risk was higher in women.

Nonetheless, the real impact of sex-related differences is still unclear. More recently, from another large review by Kostopoulou et al. [38] on sex differences in risk assessment and prevention of AF- related stroke mechanism, all-cause mortality was found higher in women. Notably, a 2-fold increase in the risk of death in AF female patients was found, compared to a 1.5-fold increase in AF male patients.

The authors underline that, in terms of predictors of thromboembolism, differences in disease severity and type of treatment, which may influence stroke risk, are not incorporated in the CHA2DS2-VASc score, since both vascular disease and hypertension are binary variables. Renal failure is also a strong predictor of stroke, but is not included in CHA2DS2-VASc. Similarly, hyperthyroidism as a cause of increased risk of stroke is more common in women but whether the thrombotic risk is higher in females compared to males is unknown. In addition, the risk for systemic embolization in thyrotoxicosis is not precisely known, and anticoagulant therapy in hyperthyroidism AF patients remains unclear [39].

Interestingly, the European Society of Cardiology guidelines [13] and a recent review on sex differences in arrhythmias by the European Heart Rhythm Association (EHRA) [40] underline these sex-specific issues and recommend offering therapeutics options to women and men, equally.

Of note, the CHA2DS2-VASc score has been evaluated as a predictor of new onset of AF, CV morbidity, and mortality in non atrial fibrillation population [41]. The authors analysed a population-based cohort of 22.179 middle-aged individuals, either with or without a history of AF (n.18.367), over a median follow-up of 15 years. The authors conclude that the CHA2DS2-VASc score is a sensitive tool for predicting new-onset AF and adverse outcomes in subjects either with or without AF.

A better net clinical benefit of NOACs compared to VKAs was demonstrated by Patti et al. [42], who evaluated the 1-year clinical outcomes in elderly (≥ 75 years) patients with AF in a prospective registry setting. This “real world” data demonstrated the better net benefit in NOACs patients, primarily due to lower rates of major bleeding, also in high-risk patients with low body mass index (BMI) and age > 85 years.

In terms of clinical presentation, as reported in the review of Poli et al. [17], the risk of AF-related complications is not different between short AF episodes and sustained forms of the arrhythmia, because of the high frequency of silent episodes which are often self-terminating. Moreover, no difference between sexes has been found for the progression of AF from paroxysmal to permanent chronic form.

As far as cardioversion and ablation is concerned, a recent clinical trial by Kuck et al. [43] assessed the association of baseline covariates with clinical outcomes in 750 patients with drug-refractory paroxysmal AF enrolled in the FIRE and ICE Trial. The authors found that female sex was associated with an almost 40% increase in the risks of primary efficacy failure and CV rehospitalization. A history of direct current cardioversion and of hypertension had a negative impact on primary efficacy failure and rehospitalization, respectively [44].

## 7. Conclusions

Female sex has been included as an independent predictor of thromboembolic risk, particularly in the elderly female population. It appears that females are less likely to receive anticoagulation therapy, possibly due to the higher age of diagnosis of AF compared to men. Female sex appears to act as a *risk modifier*, in that it seems to intensify other risk factors, particularly in VKAs patients.

Women have a lower risk to experience AF, but when they do, the stroke risk is persistently higher, even when VKAs are prescribed with anticoagulation good control.

NOACs seem to normalize sex differences, both in efficacy and in safety. As recommended by the current guidelines, NOACs are the preferred choice in NVAF patients, according to the CHA2DS2-VASc stroke risk score, which attributes 1 point to “female sex”. Anticoagulation is indicated with a 1 point or more for men and 2 or more for women. Notably, anticoagulation is not indicated for AF female patients without other risk factors. Moreover, they receive only a Class IIa-LoEB recommendation when only one additional risk factor is present. Women are frequently affected by concomitant modifiable risk factors, such as hypertension, obesity, metabolic syndrome [45, 46], thyroid dysfunction, which require accurate evaluation for an appropriate risk stratification, particularly in patients with low CHA2DS2-VASc score. When appropriate risk stratification indicates the need for anticoagulation, women should receive treatment.

A better knowledge of the different efficacy and safety profiles of NOACs compared with one another, in AF patients according to sex, could help the clinician in making the most appropriate and individualized anticoagulant therapy, either in male or in female patients.

## Figures and Tables

**Table 1:** Phase III clinical trial on non-vitamin K antagonist oral anticoagulants.

Trial	Year	Drug	Type of the study	Sample (*n*)	Mean TTR (%)	Mean CHA2DS2-VASc score	Follow-up (years)
RE-LY [27]	2009	Dabigatran	Open-label	Total patients 18,113 Men 11,514 Women 6,599	64	2.1	2.0

ROCKET-AF [28]	2011	Rivaroxaban	Double-blind	Total patients 14,264 Men 8,601 Women 5,663	55	3.5	1.9

ARISTOTLE [29]	2011	Apixaban	Double-blind	Total patients: 18,201 Men 11,785, Women 6,416	62.2	2.1	1.8

ENGAGE AF-TIMI 48 [30]	2013	Edoxaban	Double-blind	Total patients: 21,105 Men 13,065 Women 8,040	64.9	2.8	2.8

**Table 2:** Sex-based analysis of rate of stroke/SE and major bleeding studies.

Authors, year	Sample (*n*)	Type of the study	Mean CHA2DS2-VASc score	Rate of strokes/SE and major bleeding in female vs male patients	Anticoagulant therapy
Poli et al., (2013) [9]	Total patients 3,015 (aged > 80 years): Men 1,361, Women 1,654	Observational study	Females showed a higher risk score for all the classes of risk when CHA2DS2VASc was applied	Stroke: Men nr. 45 (1.3 per 100 person year) Women nr. 67 (1.6 per 100 person year) RR 1.2 (95% CI 0.8-1.8) Major bleeding: Men nr. 75 (2.2 per 100 person year) Women nr. 97 (1.4 per 100 person year) RR 1.6 (95% CI 1.1-2.3)	VKA treatment

Avgil Tsadok et al., (2015) [33]	Total patients: 15,918 Men 8,346 Women 7,572	Observational study	Men 2.6 Women 3.9	Stroke: Men HR (95% CI) 0.98 (0.78-1.23) Women HR (95% CI) 0.79 (0.56- 1.04) Major bleeding: Men HR (95% CI) 0.73 (0.64-0.84) Women HR (95% CI) 0.85 (0.71-1.01)	Dabigatran

Vinereanu et al., (2015) [34]	Total patients: 9,120 Men 5886 Women 3234	Randomized controlled trial	Men 3 (median) Women 4 (median)	Stroke/SE: Men HR (95% CI) 0.84 (0.66-1.05) Women HR (95% CI) 0.73 (0.54- 0.97) Major bleeding: Men HR (95% CI) 0.76 (0.64- 0.90) Women HR (95% CI) 0.56 (0.44- 0.72)	Apixaban vs warfarin

Camm et al., (2017) [15]	Total patients: 28, 624 Men 15 915, Women 12,709	Prospective study	Men 2.6 ± 1.5 Women 4.0 ± 1.4	Stroke/SE: Men 1.17 (CI 1.01-1.37) Women 1.62 (CI 1.41-1.87) Major bleeding: Men 0.79 (CI 1.01 - 0.95) Women 0.93 (CI 0.78 - 1.1.3)	Men: 33.8% VKA, 12.2% VKA+AP, 13.1% NOAC, 3.8% NOAC+AP, 24.9% AP, 12.2% none. Women: 37.1% VKA, 9.7% VKA+ AP, 13.9% NOAC, 3.2% NOAC+AP, 23.9% AP, 12.3% none

Law et al., (2018) [31]	Total patients: 15,292 Men 7,952 Women 7,340	Cohort study	Men 2.96 (SD 1.68) Women 4.34 (SD 1.79)	Stroke: Men HR (95% CI) 0.85 (0.65-1.12) Women HR (95% CI) 0.81 (0.63- 1.03) Major bleeding: Men HR (95% CI) 1.13 (0.73- 1.4) Women HR (95% CI) 0.89 (0.63- 1.27)	Warfarin vs DOAC

AP: Antiplatelet; CI: Confidence interval; DOAC: Direct oral anticoagulant; HR: Hazard ratio; NOAC: Nonvitamin K antagonist oral anticoagulants; RR: Risk ratio; SE: Systemic embolism; VKA: Vitamin K antagonists.

## Abbreviations

AF: Atrial fibrillation
BMI: Body mass index
CV: Cardiovascular
CVA: Cerebrovascular accidents
EU: European Union
ICH: Intracranial hemorrhage
INR: International normalized ratios
NOACs: Novel oral anticoagulants
NVAF: Nonvalvular atrial fibrillation
SE: Systemic embolism
TIA: Transient ischemic attack
TTR: Time in therapeutic range
VKAs: Vitamin K antagonist anticoagulants.
